# The GCN2 inhibitor IMPACT contributes to diet-induced obesity and body temperature control

**DOI:** 10.1371/journal.pone.0217287

**Published:** 2019-06-05

**Authors:** Catia M. Pereira, Renato Filev, Francisco P. Dubiela, Bruna B. Brandão, Claudio M. Queiroz, Raissa G. Ludwig, Debora Hipolide, Beatriz M. Longo, Luiz E. Mello, Marcelo A. Mori, Beatriz A. Castilho

**Affiliations:** 1 Department of Microbiology, Immunology and Parasitology, Escola Paulista de Medicina, Universidade Federal de São Paulo, São Paulo, Brazil; 2 Department of Physiology, Escola Paulista de Medicina, Universidade Federal de São Paulo, São Paulo, Brazil; 3 Department of Psychobiology, Escola Paulista de Medicina, Universidade Federal de São Paulo, São Paulo, Brazil; 4 Department of Biophysics, Escola Paulista de Medicina, Universidade Federal de São Paulo, São Paulo, Brazil; 5 Brain Institute, Universidade Federal do Rio Grande do Norte, Natal, Brazil; 6 Department of Biochemistry and Tissue Biology, UNICAMP, Campinas, Brazil; National Cancer Institute, UNITED STATES

## Abstract

IMPACT, a highly conserved protein, is an inhibitor of the eIF2α kinase GCN2. In mammals, it is preferentially expressed in neurons. Knock-down of IMPACT expression in neuronal cells increases basal GCN2 activation and eIF2α phosphorylation and decreases translation initiation. In the mouse brain, IMPACT is particularly abundant in the hypothalamus. Here we describe that the lack of IMPACT in mice affects hypothalamic functions. *Impact*^-/-^ mice (*Imp*-KO) are viable and have no apparent major phenotypic defect. The hypothalamus in these animals shows increased levels of eIF2α phosphorylation, as expected from the described role of IMPACT in inhibiting GCN2 and from its abundance in this brain region. When fed a normal chow, animals lacking IMPACT weight slightly less than wild-type mice. When fed a high-fat diet, *Imp*-KO animals gain substantially less weight due to lower food intake when compared to wild-type mice. STAT3 signaling was depressed in *Imp*-KO animals even though leptin levels were identical to the wild-type mice. This finding supports the observation that *Imp*-KO mice have defective thermoregulation upon fasting. This phenotype was partially dependent on GCN2, whereas the lean phenotype was independent of GCN2. Taken together, our results indicate that IMPACT contributes to GCN2-dependent and -independent mechanisms involved in the regulation of autonomic functions in response to energy availability.

## Introduction

The protein IMPACT was first identified as the product of an imprinted gene in mice, containing a so-called "ancient domain" due to its strong similarity with bacterial proteins [1]. Another domain of the IMPACT protein and of its yeast ortholog, Yih1, shares sequence similarity with a region present in GCN2, a kinase that regulates protein synthesis. Several studies performed in mammalian cells as well as in yeast have indicated that IMPACT/Yih1 inhibits the activity of GCN2 [2–7].

GCN2 is one of the four kinases in mammals, activated by different stress conditions, that specifically phosphorylate the translation initiation factor eIF2 at residue Ser51 of its alpha subunit, triggering a program known as the integrated stress response (ISR) (reviewed in [8, 9]). This regulatory program is fundamental for several aspects of normal mammalian physiology, as determined from several models of knock-out mice devoid of one or more of the eIF2 kinases, GCN2, PERK, PKR or HRI, or knock-in animals, where the Ser51 residue in eIF2α was mutated to a non-phosphorylatable residue (reviewed in [9–11]). Mammalian GCN2 controls feeding behavior and memory, immune system regulation and cancer cell survival (reviewed in [12]).

At each round of initiation, eIF2 delivers the initiator Met-tRNA^Met^ to the 40S ribosomal subunit, as a ternary complex with GTP. Upon recognition of the initiator AUG codon in the mRNAs, the eIF2-bound GTP is hydrolyzed and eIF2-GDP is released. For another round of initiation, GDP must be exchanged for GTP by eIF2B. Phosphorylated eIF2α acts as an inhibitor of eIF2B, therefore affecting the rates of initiation of protein synthesis. The ratio of phosphorylated/unphosphorylated eIF2α in the cells dictates the extent of translation inhibition [13]. While this event inhibits general translation initiation, it facilitates the translation of certain mRNAs that encode proteins required for the cells to recover from the initial insult. The immediate downstream target of increased eIF2α(P) levels is the increased translation of the mRNA encoding for ATF4 in mammals. ATF4 is a transcriptional factor that regulates a large number of genes, whose products help cells to cope with and recover from the initial stress condition that led to the activation of this pathway [9]. A mild and controlled ISR protects cells from injuries, whereas strong and sustained stress may result in cell death.

GCN2 is activated by the binding of uncharged tRNAs that accumulate in cells when amino acids are scarce. This binding induces a conformational change in GCN2 that leads to its activation and autophosphorylation at a Threonine residue in the catalytic domain [14–17]. [reviewed in [18]].

The activation of GCN2 also requires its association with its effector protein, GCN1, through an N-terminal domain called RWD [19–22]. A RWD domain is found in other proteins, such as the protein IMPACT, in mammals, and its yeast ortholog, Yih1 (reviewed in [18]). Studies in yeast and in mammals have shown that Yih1 and IMPACT act as inhibitors of GCN2 due to a competition for the binding to GCN1 [2–5, 7]. In *C*. *elegans*, IMPACT also functions as a GCN2 inhibitor, limiting lifespan [23].

Mammalian IMPACT is preferentially expressed in neurons [24]. IMPACT abundance increases drastically upon neuronal differentiation, both in neuronal cell lines as well as in mice [7]. The knock-down of IMPACT expression in differentiating neuronal cells leads to increased basal levels of active GCN2 and of phosphorylated eIF2α, decreased translation initiation and increased expression of ATF4 [7]. In the neuronal-like N2a cells IMPACT promotes neuritogenesis, while GCN2 is a strong inhibitor of neurite outgrowth [7].

In the central nervous system, neurons with high IMPACT expression are found scattered throughout the brain, but the hypothalamus is strikingly rich in neurons that overexpress IMPACT [24]. These observations indicate that IMPACT may be relevant in the central control of physiological homeostatic mechanisms.

Given the relevance of regulation of protein synthesis for neuronal functions and: i) the physiological roles of GCN2 in behavioral and metabolic events; ii) our previous evidence that IMPACT modulates GCN2 activity; iii) the high levels of IMPACT in neurons; and iv) the extreme abundance of IMPACT in the hypothalamus, we set out to study the function of IMPACT in mammals, by developing an *Impact*^-/-^ mouse (Imp-KO). We describe here the initial characterization of these animals. We show that the levels of eIF2α(P) are increased in the hypothalamus of *Impact*^-/-^ mice, as expected from the role of this protein as an inhibitor of GCN2. Mice lacking IMPACT are leaner than wild-type mice, especially when fed a high-fat diet. This phenotype was due to lower food ingestion and was observed in mice lacking both IMPACT and GCN2, suggesting independence of IMPACT’s role as a GCN2 inhibitor. We also show that animals lacking IMPACT have a defect in the control body temperature in response to starvation, and this seems to be partially dependent on GCN2. The results shown here indicate that the evolutionarily conserved IMPACT protein is involved in the maintenance of energy homeostasis in mice.

## Materials and methods

### Animals

This study was conducted under protocols approved by the Animal Care and Use Ethics Committee of the Universidade Federal de São Paulo, in accordance with the Guide for Care and Use of Laboratory Animals adopted by the National Institutes of Health (Permit numbers: CEP-1652/2010; CIBio-17/2012; CEUA-2740040414). Mice were in the C57Bl/6J background. All data shown here were obtained from male mice. Animals were maintained in a 12h/12h light-dark cycle, at 23°C, with water and food *ad libitum*, except when otherwise indicated.

*Impact-*Lox conditional animals were obtained by Ozgene, Inc. (Australia), by inserting LoxP sites in the intron between exons 2 and 3, and in the intron between exons 4 and 5 of the *Impact* gene. The Neo^R^ determinant, under the control of the PGK promoter, was inserted in the intron between exons 4 and 5. These mice were then bred to general CRE deleter animals (Cre-EIIa) (Jackson Laboratory). Upon CRE-mediated recombination, exons 3 and 4 were removed; splicing of exon 2 to 5 causes a frame-shift mutation that creates an early stop codon, with the resulting peptide having the sequence MAEEEVGNSQRQSEEIEAMAAIYGEEWCVIDENAKIFCIRVTDFMDDPKWTLCLQPQHG*, with the underlined residues derived from the frame shift. Cre-Ella was then removed by crossings with C57Bl/6J mice. Routine genotyping of the established *Imp*-KO strain was performed by PCR of tail DNA, using the following primer pairs: P1 (5’-CCTGACTCTGGCTTCATAATACTG-3’) and P4 (5’-GGATCTACTGCACTGCCTACC) amplifies a fragment of 605bp of the knock-out allele and of 1,421bp of the wild-type allele; P8 (5’-GATGTTGCCAAGTGAGTACCCG-3) and P9 (5’-CATATATCTCCTCAAGGCTGTTCG-3’) amplifies a fragment of 550bp of the wild-type locus only. *Gcn2*-KO animals in the C57Bl/6J background have been described [7, 25].

### Diets

Mice were between 6–8 week old at the beginning of the experiments. Individual animal weight was determined each week at 9 a.m., and food ingestion determined in the same day, for each animal cage containing 3–5 animals. Normal chow was 22% protein and 4% fat (Nuvilab, Brazil); high-fat diet contained 20% protein and 30% fat (Pragsoluções, Brazil).

### Assays

For glucose tolerance tests, mice were fasted overnight and at 9 a.m. injected intraperitoneally with glucose (10 μl/g of body weight of 20% glucose solution in saline). Insulin tolerance tests were performed at 9 a.m., after a 2-hour fasting, with the intraperitoneal administration of insulin (0.75 U/kg body weight, for animals under a normal chow, or 1.25 U/kg of body weight, for animals fed a high-fat diet). Glucose values were measured from tail blood using a glucometer (Accu-Chek Active, Roche). Serum leptin concentration was determined by ELISA (B&D Systems).

### Temperature measurements

Body temperature was measured using implanted transponders (IPTT-300, BMDS), except when otherwise specified, at the indicated time of day. For the 4°C environmental temperature, the animals were placed in a 4°C room, at 9 a.m., with lights on.

### Indirect calorimetry

O_2_ consumption and CO_2_ production were determined by indirect calorimetry in a continuous monitoring system (Columbus Instruments, Inc.). Eight-week-old mice (8 wild-type and 8 *Imp*-KO) were monitored during 24h, in the presence or absence of food.

### Organ extracts and immunoblots

Organs were removed and extracts immediately prepared in buffer containing 50 mM Tris-HCl, pH 7.5, 150 mM NaCl, 1 mM EDTA, 10 mM MgCl_2_, 1% Triton X-100, 1 x protease inhibitor Halt EDTA-free cocktail (Pierce), 10 mM sodium fluoride, 1 mM sodium orthovanadate, 17.5 mM sodium ß-glycerophosphate and 6 mM sodium pyrophosphate. Extracts (20–50 μg total protein) were run on 12% SDS-PAGE (7% SDS-PAGE for GCN2 detection) and proteins transferred to nitrocellulose membranes (Hybond C, GE Life Sciences). After blocking with 5% low fat milk, the membranes were incubated in TBS-0.1% Tween 20, 5% BSA, with antibodies against the indicated proteins, followed by the appropriate secondary antibodies conjugated to HRP, and detection with ECL reagent (GE Life Sciences). Quantification of signals was performed using ImageJ. Antibodies used were: anti-STAT3 (Santa Cruz) and anti-STAT3-P (Cell Signaling); anti-eIF2α and anti-eIF2α(P) (Life Technologies); anti-IMPACT [4, 24] and anti-GCN2 [24].

### Immunohistochemistry

One hour after saline or leptin injection (3μg/g body weight), the animals were deeply anaesthetized and perfused through the heart with 50 mL of phosphate-buffered saline (PBS) followed by 200 mL of 4% paraformaldehyde at 4°C. Brains were removed and cryoprotected in 30% sucrose in PBS for 24h. Coronal brain cryostat sections (30-μm thick) were made according to the stereotaxic coordinates of the mouse brain atlas. Sections of the hypothalamus were selected (5 per animal) to verify the labeling pattern of STAT3 activation by immunohistochemistry using an antibody directed to the phosphorylated form of STAT3. Free-floating sections were washed in 0.02M PBS and washed with 0.5% sodium borohydride (20 min.), 0,3% NaOH + 0.3% H_2_O_2_ (20 min.), 0,3% glycine (10 min.) and 0.03% SDS (10 min.); all the solutions were prepared in 0.02M PBS. Incubation in blocking solution (4% normal goat serum, 0.4% Triton-X 100, 1% BSA in 0.02M PB) was performed for 30 minutes, followed by overnight incubation with the rabbit anti-phospho STAT3 antibody (1:200; Cell Signaling, D3A7) diluted in blocking solution. The sections were then washed and incubated with biotinylated secondary antibody against rabbit IgG (1:600, Vector, Burlingame, CA, USA) for 2 h. After incubation, the sections were washed in PBS and incubated in the ABC kit solutions (Vectashield, Vector, Burlingame, CA, EUA) for 1h30min. The sections were stained with diaminobenzidine (DAB, Sigma-Aldrich Corporation, St. Louis, EUA), mounted on slides and sealed with coverslips.

### RT-qPCR

Total RNA was isolated using TRizol Reagent (Invitrogen) according to the manufacturer’s specifications with slight modifications. Briefly, samples were homogenized in TRizol Reagent, mixed with chloroform (5:1 v/v sample /chloroform) and centrifuged at 12,000 x g for 15 min at 4°C. The aqueous phase containing total RNA was collected, diluted 1:1 (v/v) in isopropanol, let stand overnight at -20°C and precipitated in the next day by centrifugation at 12,000 x g for 10 min at 4°C. Pellets were washed with 75% ethanol, dried and resuspended in nuclease-free water. cDNA was synthesized from 1 μg of total RNA by using High Capacity cDNA Reverse Transcription Kit (Applied Biosystems) and random primers. 0,15-μL of cDNA was used in a 6-μL qPCR (Maxima SYBR Green qPCR Master Mix; Fermentas, Glen Burnie, MD) containing 250 nM primers for Ucp1 (F—CTGCCAGGACAGTACCCAAG, R—TCAGCTGTTCAAAGCACACA) and Adrb3 (F—GTCATCTACTGCCGCAGCCC, R—CCTGTTGAGCGGTGGACTCTG). Reactions were run in duplicate using the CFX384 Touch Real-Time PCR Detection System (Bio-Rad) under the following conditions: 50°C -2 min, 95°C -10 min, and 40 cycles of 95°C -15s, 60°C -20s, and 72°C -30s. Dissociation protocols were conducted after every run to check for primer specificity. To obtain relative expression values, we calculated the 2-ΔCt parameter for each individual sample using Ct values of 36b4 endogenous control as a reference (F-TTTGACAACGGCAGCATTTA, R-CCATTGATGATGGAGTGTGG).

## Results

### Animals lacking IMPACT are viable and have increased phosphorylation of eIF2α in the hypothalamus

**Fig 1:**
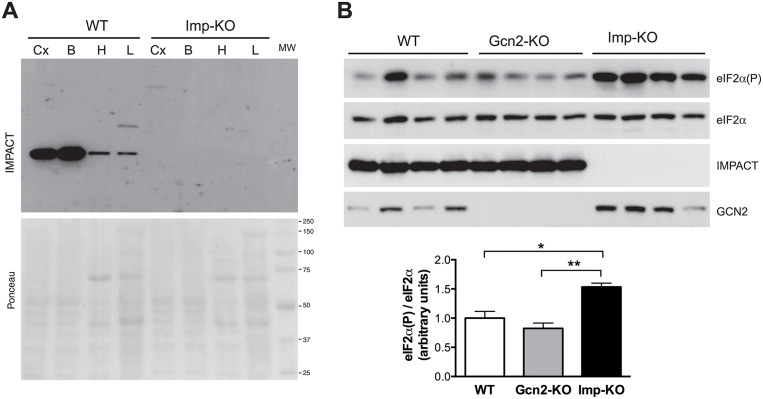
Absence of IMPACT increases eIF2α phosphorylation in the hypothalamus. **(A)** Western blot of extracts of the cortex (Cx), remaining brain parts (B), heart (H) and liver (L) of wild-type (WT) and *Impact*^-/-^ animals (Imp-KO), using antibodies against IMPACT (upper panel). The Ponceau stained membrane is shown in the bottom panel with the molecular mass standards indicated on the right (in kDa). **(B)** Western blots (top panel) of extracts of the hypothalamus isolated from wild-type animals (WT), animals lacking GCN2 (Gcn2-KO) and animals lacking IMPACT (Imp-KO) (four animals each), using antibodies against the phosphorylated form of eIF2α (eIF2α(P)) and total eIF2α (eIF2α), IMPACT and GCN2. The levels of phosphorylated eIF2α were normalized against total eIF2α for each animal, and the mean ± SEM for each genotype is shown in the graph (bottom panel). * P<0.05; **P<0.005 (Student’s t-test). Where not indicated, the difference between genotypes was not statistically significant.

*Impact*^-/-^ homozygote (*Imp*-KO) mice were viable, born under normal Mendelian ratio and fertile. To determine whether the genomic alteration resulted in the complete lack of the protein IMPACT, different organs were obtained from these animals and subjected to immunoblots using anti-IMPACT antibodies. As shown in Fig 1A, no IMPACT protein was detected in the heart, liver or brain parts of the *Imp*-KO mice.

We have previously shown that IMPACT is highly abundant in the hypothalamus. Based on previous *in vitro* data indicating that IMPACT is an inhibitor of GCN2, we then investigated the levels of eIF2α phosphorylation in the hypothalamus of *Imp*-KO animals. In addition, we included in this study hypothalamic extracts obtained from *Gcn2*-KO animals. Four animals were analyzed for each genotype (Fig 1B). As expected, in *Imp*-KO mice there was a striking increased ratio of eIF2α(P)/eIF2α in the hypothalamus, while in *Gcn2*-KO mice eIF2α(P) was somewhat lower than in wild-type mice, and significantly lower than in *Imp*-KO animals. Thus, the lack of IMPACT results in increased basal levels of hypothalamic eIF2α phosphorylation, likely due to the increased basal activation of GCN2.

### *Imp*-KO mice are leaner than wild-type mice

**Fig 2:**
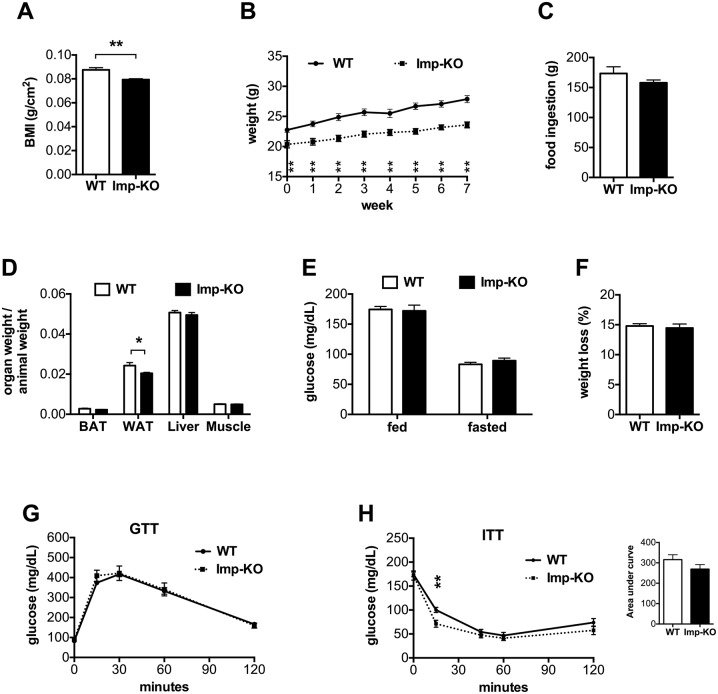
Animals lacking IMPACT are leaner than wild-type mice. **(A)** body mass index, determined from *Imp*-KO mice and their wild-type littermates; **(B)** weight gain on regular chow; eight-week old wild-type (n = 10) and *Imp*-KO (n = 8) animals were fed with regular chow and weight determined at weekly intervals for seven weeks; weight differences between the two genotypes were statistically significant at all times; **(C)** total food intake for the seven weeks measured for each cage, and normalized for the number of animals in each; **(D)** organ weights normalized against each animal weight, at week 10; **(E)** glucose levels in fed and fasted (18h) animals; **(F)** weight loss after an 18h fasting, indicated as percentage of initial weight; **(G)** glucose tolerance and **(H)** insulin tolerance tests were performed at weeks 8 and 9, respectively, of the same diet. All values are mean ± SEM. * P<0.05; **P<0.005 (Student’s t-test). Where not indicated, the difference between genotypes was not statistically significant.

Homozygote progeny from heterozygote crossings were initially analyzed for a series of basic behavioral tests and phenotypic aspects. A slight weight difference was noticed in *Imp*-KO mice relative to their wild-type littermates and their body mass index is shown in Fig 2A. All subsequent analyses were then performed with the established homozygous strain. We measured weight gain for 7 weeks in animals fed a normal diet. Throughout this period, *Imp*-KO animals maintained a lower weight compared to wild-type animals (Fig 2B). Food intake measurements suggested that the mice lacking IMPACT ingested less chow than wild-type mice (Fig 2C). At week 11 of this feeding regimen, the animals were sacrificed and the weight of different organs determined relative to body weight (Fig 2D). The main difference was in the white fat depots. Glucose levels in mice lacking IMPACT did not differ significantly from wild-type mice in both fed and fasting conditions (Fig 2E). Upon fasting, Imp-KO mice lost the same percentage of the initial weight as the wild-type animals (Fig 2F). Prior to sacrifice, the same group of mice was subjected to glucose and insulin tolerance tests (Fig 2G and 2H). No difference was observed between the two genotypes in glucose tolerance. For insulin tolerance, the animals lacking IMPACT seem to be slightly more sensititve to insulin.

**Fig 3:**
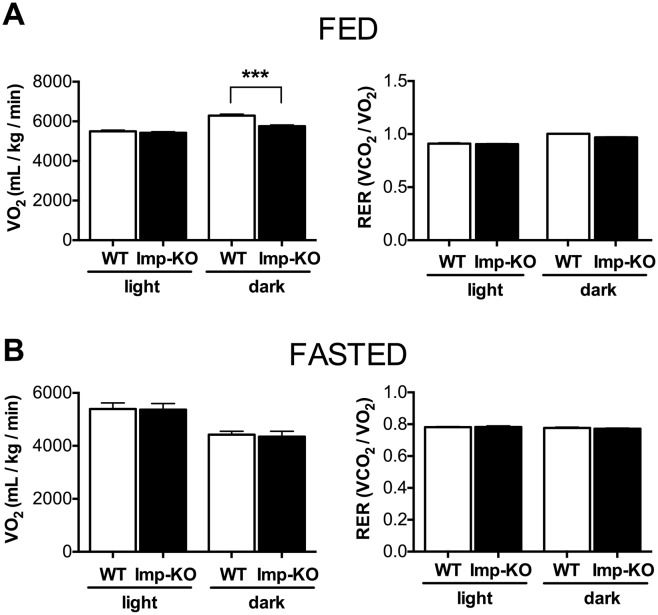
Energy expenditure. Mice (n = 8 each genotype; eight-week-old) were subjected to measurements of the volume of O_2_ consumption and CO_2_ production by indirect calorimetry in a Columbus system for **(A)** 24 hours with animals maintained in normal chow (fed) or **(B)** 18 hours of fasting. Oxygen consumption (VO_2_) and respiratory exchange ratio (RER) are shown for the light and dark periods for fed and fasted animals. Data are mean ± SEM. ***P<0.0005 (Student’s t-test). Where not indicated, the difference between genotypes was not statistically significant.

Energy expenditure was analyzed in 2-month old animals maintained in a normal chow and in fasting animals (Fig 3). The amount of oxygen consumption and the respiratory exchange ratio were determined by indirect calorimetry during the light and dark periods. Animals lacking IMPACT had lower consumption of oxygen during the dark period, relative to the wild-type mice (Fig 3A). This difference was not observed in fasted animals (Fig 3B). Weight loss due to an 18 h fasting period was similar for the two groups (Fig 2F). These results indicate that reduced body weight and fat mass in *Imp*-KO animals are not due to increased energy expenditure.

Previous reports have indicated that increased phosphorylation of eIF2α by intracerebral administration of salubrinal results in increased deep slow wave sleep and decreased awake periods [26]. Since this is also autonomically controlled by the hypothalamus and could affect feeding behavior, we subjected these animals to analyses of sleep patterns. No difference was found in the sleep-wake architecture of mice lacking IMPACT in comparison to wild-type animals (S1 Fig). In addition, there was no significant difference in home cage activity between the two groups (S2 Fig).

### The lack of IMPACT protects mice from high-fat diet-induced obesity

**Fig 4:**
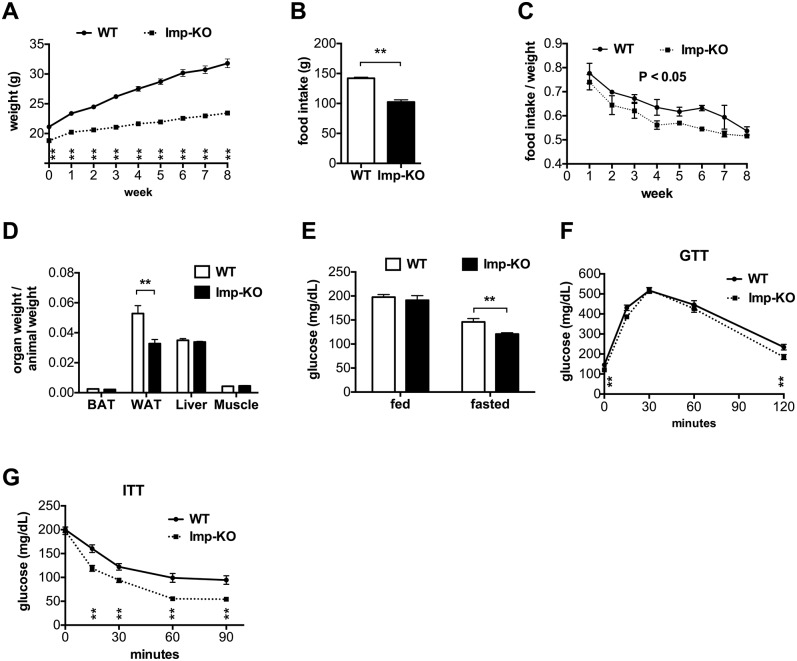
The lack of IMPACT protects mice from obesity induced by a high-fat diet. Six to eight-week old wild-type (n = 10) and *Imp*-KO (n = 10) animals were fed with a high-fat diet for 12 weeks. **(A)** Individual animal weight determined weekly. **(B)** Total food intake per animal for the eight week period. **(C)** Food intake normalized per weight at weekly intervals for the eight weeks. **(D)** At week 13, animals were sacrificed and their organs weighted, and normalized against total body weight for each animal. **(E)** Glucose levels in fed and fasted (18h) animals. Glucose **(F)** and insulin **(G)** tolerance tests were performed at week 11 and 12 respectively of the same diet. The values represent mean ± SEM. * P<0.05; **P<0.005 (Student’s t-test, except for panel C, where two-way ANOVA was used). Where not indicated, the difference between genotypes was not statistically significant.

We then asked whether the lack of IMPACT would protect mice from obesity induced by a high-fat diet (HFD). As shown in Fig 4A, the difference in weight gain was striking, with the *Imp*-KO mice maintaining a lean phenotype, while wild-type mice gained substantial weight as expected. Total food intake during the eight weeks was substantially lower for the *Imp*-KO mice (Fig 4B). This diference was also significant when the amount of ingested chow was normalized against the animal weight, as shown for weekly measurements (Fig 4C). We also included *Gcn2*-KO animals in the high-fat diet regimen and no difference in weight gain was observed relative to the wild-type mice (S3 Fig).

To determine body mass composition, after 13 weeks of the HFD feeding regimen, the animals were sacrificed, and their organs weighted. When normalized for the individual body weight, the major difference between the wild-type and *Imp*-KO mice was in the white fat depots (Fig 4D).

Glucose and insulin tolerance was evaluated at week 11 and 12, respectively, of the HFD feeding regimen. Fed glucose levels were identical for both genotypes (Fig 4E). After an 18h fasting, however, *Imp*-KO mice had significantly lower blood glucose levels. The *Imp*-KO mice responded better than the wild-type mice to glucose administration (Fig 4F). Consistent with the leaner phenotype and the lower glucose levels, the *Imp*-KO mice were significantly more sensitive than wild-type mice to an insulin challenge (Fig 4G).

### Central STAT3 signaling is defective in animals lacking IMPACT

**Fig 5:**
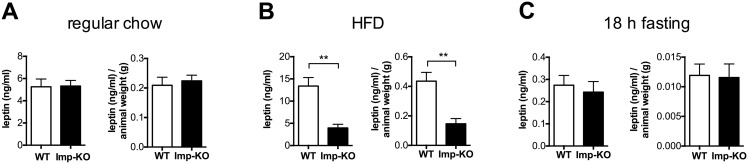
Leptin levels in mice lacking IMPACT. Total serum leptin and leptin normalized by the animal weight are shown for animals maintained in normal diet **(A)**, maintained in a high-fat diet (HFD) for 12 weeks **(B)**, and for animals fed normal diet but fasted for 18 hours **(C)**. n = 6 for each genotype. **P<0.005 (Student’s t-test). Where not indicated, the difference between genotypes was not statistically significant.

**Fig 6:**
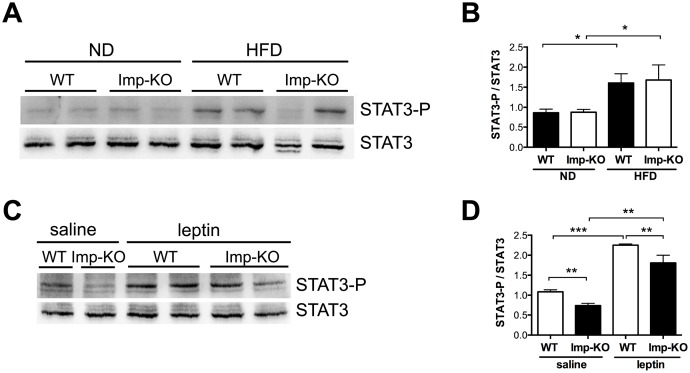
Hypothalamic STAT3 signaling. **(A)** Representative immunoblots of protein extracts from the hypothalamus isolated from two animals from each group shown in Figs 2 and 4 using antibodies against the phosphorylated form of STAT3 (STAT3-P) (upper panel); after stripping, the same membrane was used for incubation with antibodies against total STAT3 (lower panel). **(B)** Quantification of STAT3-P/STAT3 from duplicate immunoblots performed as in (A) for hypothalamic extracts from 4 animals of each diet and genotype. **(C)** hypothalamic STAT3 signaling 1 hr after intraperitoneal administration of saline or leptin (3μg/g body weight), determined by immunoblots as described in (A). **(D)** quantification of STAT3-P/STAT3 from duplicate immunoblots performed as in (A) for hypothalamic extracts of 4 animals of each genotype for the saline and for the leptin injection. *P<0.05; **P<0.005 (Student’s t-test). When not indicated, the difference between genotypes was not statistically significant.

Serum leptin levels were determined from the blood collected at the end of the normal chow and HFD feeding experiments, when animals were sacrificed. While no difference in leptin levels was observed under normal chow (Fig 5A), in the high-fat diet, *Imp*-KO mice had much less leptin than wild-type animals (Fig 5B). This difference was observed even when leptin concentrations were normalized against total body weight. These data are consistent with the decreased white fat depots observed in *Imp*-KO mice. Fasting levels of leptin were similar between the two groups (Fig 5C). The analysis of the hypothalamic STAT3 signaling in the mice maintained in normal chow suggested no difference between the genotypes (Fig 6A and 6B).

We then studied the STAT3 signaling in response to the administration of identical doses of leptin in mice that were maintained in regular chow but fasted for 18 h prior to intraperitoneal leptin (3μg leptin/g body weight) injection. One hour after the injection, animals were sacrificed and the hypothalamic STAT3 phosphorylation determined (Fig 6C and 6D). Interestingly, in the control experiment with saline administration mice lacking IMPACT showed significantly lower STAT3-P levels compared to wild-type animals even though the leptin levels were similar between the two genotypes (Fig 5C). The *Imp*-KO mice responded to leptin administration, but maintained a lower level compared to the wildtype mice. These data taken together indicate that *Imp*-KO mice have a defective hypothalamic STAT3 signaling.

### Feeding-dependent thermoregulation is affected in the *Imp*-KO mice

**Fig 7:**
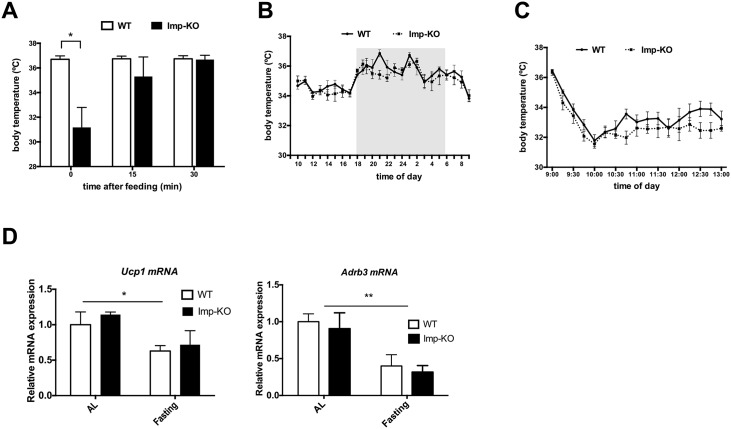
Defective body temperature control of *Impact*^-/-^ animals. Temperature was measured at the specified times and conditions by telemetry from sub-cutaneously implanted transmitters (n = 5 of each genotype). **(A)** Animals were fasted overnight and fed at 8:30am. **(B)** Animals were maintained in normal conditions; **(C)** Animals were transferred to a 4°C room at 9 am. *P<0.05; ***P<0.0005 (Student’s t-test). When not indicated, the difference between genotypes was not statistically significant. **(D)** mRNA expression of Uncoupling protein 1 (Ucp1) and beta-3 adrenergic receptor (Adrb3) in brown adipose tissue from animals after overnight fasting, or fed *ad libitum* (AL) (n = 3 animals for each group), as assessed by qPCR. *P < 0.05; **P < 0.01 (Two-way ANOVA with Bonferroni post hoc test).

During the course of the experiments described above, we noticed that *Imp*-KO mice were significantly colder after prolonged fasting. We then measured the body temperature of these mice with a sensor implanted subcutaneously (Fig 7A). After 18h of fasting, mice lacking IMPACT showed a drastically reduced body temperature. After refeeding, the temperature increased quickly, reaching normal values after 30 minutes.

Under normal feeding conditions, during a 24 h monitoring, no significant difference was found relative to the wild-type animals, although a slightly lower temperature was detected at the beginning of the dark period (Fig 7B). The *Imp*-KO mice were also capable of adjusting their body temperature in response to a cold environment, but again a slightly lower temperature was observed (Fig 7C). The expression of Uncoupling protein 1 (Ucp1) and of beta3-adrenergic receptor mRNAs in brown adipose tissue isolated from fed and fasting Imp-KO mice were similar to those of the wildtype animals, implying that this main thermogenic mechanism was not affected by the lack of IMPACT (Fig 7D).

### Phenotypic dependence on GCN2

**Fig 8:**
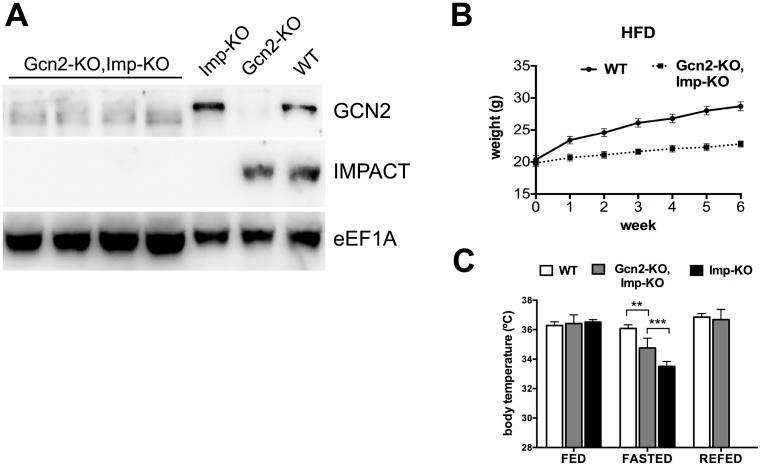
Phenotypes of double knock-out animals. **(A)** Western blot of extracts of the cortex of four double knock-out animals (*Gcn2*-KO,*Imp*-KO), one *Imp*-KO animal, one *Gcn2*-KO animal and a wild-type mouse, using antibodies against IMPACT and against GCN2; eEF1A was used as a loading control; **(B)** Wild-type (n = 12) and *Gcn2*-KO,*Imp*-KO (n = 7) mice were submitted to a high-fat diet and weight determined as described in Fig 4; **(C)** temperature after overnight fasting was determined in wild-type (n = 5), *Gcn2*-KO,*Imp*-KO (n = 8) and *Imp*-KO (n = 5) mice, using a rectal probe thermometer.

Recent data from our group, using both mammalian cells and yeast, have suggested that IMPACT/Yih1 may have other functions in addition to acting as a GCN2 inhibitor [7, 23, 27]. To determine whether the phenotypes observed for the *Imp*-KO animals were dependent on GCN2, we obtained double knock-out mice (*Imp*-KO,*Gcn2*-KO) (Fig 8A). These animals were then subjected to a high-fat diet. As shown in Fig 8B, the absence of GCN2 did not revert the lean phenotype described for the *Imp*-KO mice maintained in a high-fat diet, indicating that it is independent of GCN2. The *Imp*-KO,*Gcn2*-KO mice were also studied for their body temperature after prolonged fasting (Fig 8C). In this case, the phenotype observed for the *Imp*-KO was partially dependent on GCN2.

**Fig 9:**
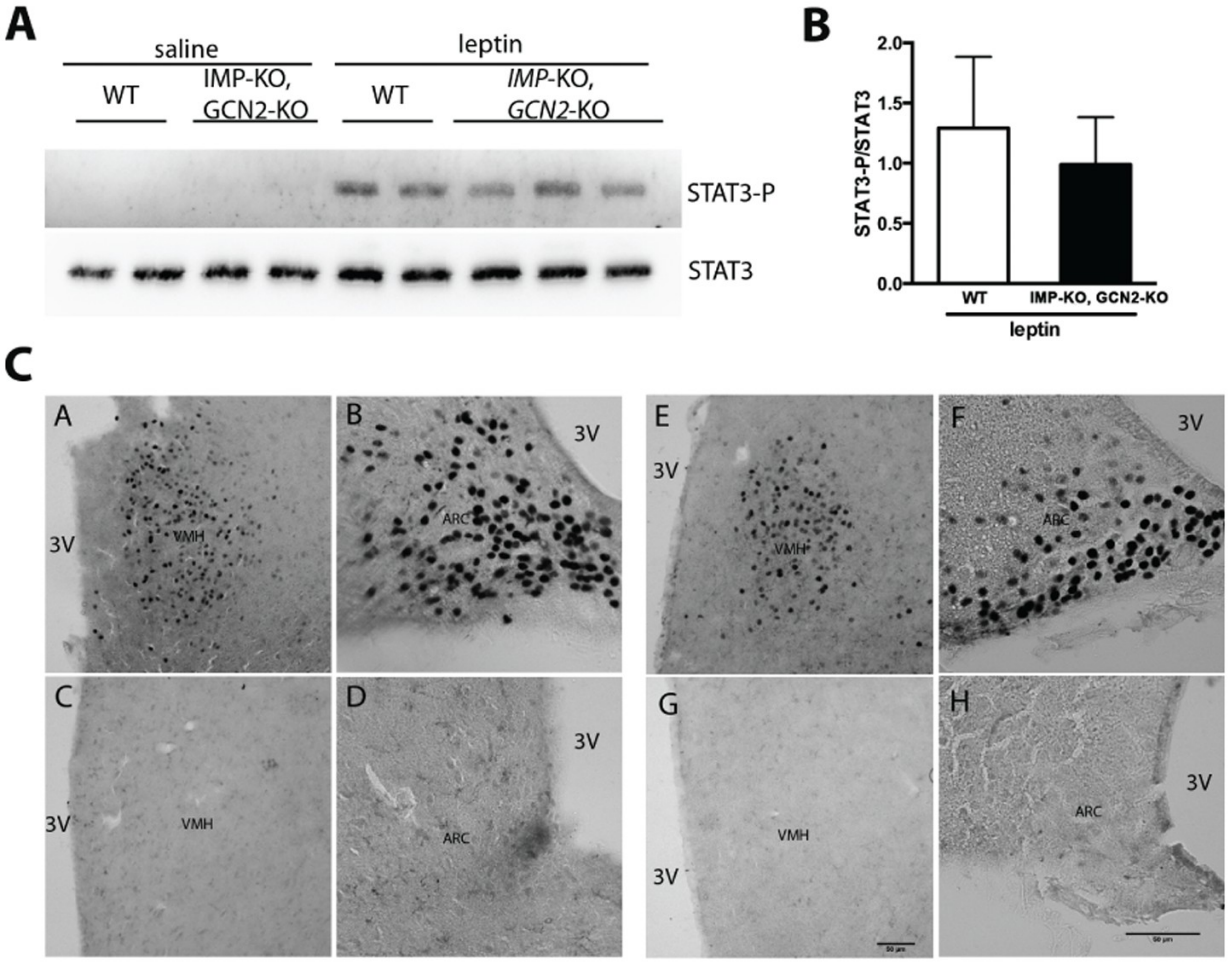
STAT3 activation in the hypothalamus of *Gcn2*^-/-^,*Impact*^-/-^ mice. **(A)** Representative immunoblots of hypothalamic extracts of animals after intraperitoneal administration of saline or leptin using antibodies against the phosphorylated form of STAT3 (STAT3-P) (upper panel); after stripping, the same membrane was used for incubation with antibodies against total STAT3 (lower panel). Each group is composed of 4 animals, except the *IMP*-KO, *GCN2*-KO leptin injected group which is formed by 5 animals. **(B)** Quantification of STAT3-P/STAT3 from triplicate immunoblots performed as in (A) for hypothalamic extracts from 4 or 5 animals of each group. **(C)** Immunohistochemical labeling patterns for phosphorylated form of STAT3 (STAT3-P) in the ventromedial nucleus (VHM) and arcuate nucleus (ARC) after leptin (A,B and E, F) or saline intraperitoneal injection (C, D and G, H) in WT (A, B, C, D) and *IMP*-KO, *GCN2*-KO (E, F, G, H) mice. The panels show representative slices of the four groups composed of three animals each.

The STAT3 signalization was also analyzed in *Imp*-KO, *Gcn2*-KO mice. Although not statisticaly significant in this experiment, the *Imp*-KO, *Gcn2*-KO mice responded to leptin administration in a lower level compared to the wildtype mice, as observed for *IMP*-KO mice (Fig 9A and 9B). These data indicate that the defective hypothalamic STAT3 activation observed in *IMP*-KO mice is independent of GCN2. We then studied whether the absence of IMPACT would affect the pattern of STAT3 phosphorylation in the hypothalamus, thus interfering in the homeostatic response to leptin. Immunohistochemistry of brain slices using anti-STAT3-P specific antibody shows no remarkable difference in STAT3 activation between the wildtype and *IMP*-KO, *Gcn2*-KO mice in the ventromedial or arcuate nuclei, the main leptin-responsive hypothalamic nuclei (Fig 9C).

## Discussion

We have shown here that the protein IMPACT contributes to the weight gain and body temperature homeostasis in mice.

From previous results of this group indicating that IMPACT is an inhibitor of GCN2 and that it is extremely abundant in the hypothalamus, we expected that the hypothalamic levels of phosphorylated eIF2α would be higher in mice lacking IMPACT. Indeed, this was clearly observed. The contribution of GCN2 to hypothalamic eIF2α(P) is evident, as shown by the decreased eIF2α(P)/eIF2α ratio observed in mice lacking GCN2. Thus, the absence of IMPACT, and therefore, the increased levels of basal GCN2 activity may account for the increased eIF2α phosphorylation observed in the *Imp*-KO animals. This is the first animal model in which eIF2α phosphorylation is physiologically increased.

The hypothalamus is responsible for a large array of responses that adjust metabolic and behavioral responses to the immediate needs in order to maintain organismal homeostasis. Such responses include feeding behavior in response to leptin, produced mainly in the white adipose tissue. In the hypothalamus, the expression of IMPACT is homogeneously high, except for the ventromedial hypothalamic nucleus [24]. It has been recently described that increased eIF2α phosphorylation in the hypothalamic arcuate nucleus obtained by administration into the third ventricle of salubrinal, a drug that inhibits the activity of eIF2α phosphatases therefore increasing levels of eIF2α(P), induces a decrease in food ingestion [28]. The arcuate nucleus is rich in neurons with high expression of IMPACT [24]. The arcuate nucleus is also rich in neurons expressing the leptin receptor, which by means of JAK2, phosphorylates STAT3. Phosphorylated STAT3 migrates to the nucleus where it acts as a transcriptional activator of genes that regulate feeding behavior, for example, pro-opiomelanocortin (POMC) or neuropeptide Y (NPY) in specific neurons. Data shown here suggest that IMPACT may regulate the sensitivity on this brain region to changes in leptin levels.

*Gcn2*-KO animals did not differ from wild-type animals in weight gain and food ingestion in high-fat diet. This is in agreement with a recent report showing that the lentiviral-mediated knock-down of GCN2 in the arcuate nucleus did not alter regular chow intake [28]. It seems then that it is the increase in eIF2α(P) levels that inhibits food intake. A downstream effect of increased levels of phosphorylated eIF2α is the promotion of translation of the message encoding the transcriptional factor ATF4. The upregulation of the stress-responsive hormone GDF15 mediated by increased ATF4 seems to mediate food aversion, as recently described, in a mechanism that involves the brainstem [29]. It is possible then that IMPACT, by controlling the levels of active GCN2 and therefore of eIF2α(P), regulates feeding behavior by multiple mechanisms. However, the results obtained with mice lacking both GCN2 and IMPACT seem to suggest that this is not a direct correlation, since these animals showed identical lean phenotypes as mice lacking only IMPACT. It is likely then that IMPACT affects also other pathways, in addition to GCN2, that ultimately result in decreased food intake.

On the other hand, the effect of the lack of IMPACT in body temperature control under starvation conditions was partially dependent on GCN2. Overexpression of the ATF4 in the third ventricle provokes some of the effects we observed in the *Imp*-KO mice, including decreased body temperature [30]. Thus, IMPACT affects temperature control in a GCN2-dependent mode probably through regulation of ATF4 expression. The GCN2-independent mechanism by which IMPACT affects temperature control may involve other pathways. We showed here that the lack of IMPACT does not involve the main thermogenic driver, UCP1. Alternatives that could result in the observed phenotype could involve other regulators of brown adipose tissue heat generation, or an impairment in shivering thermogenesis. Further studies are required to unravel the role of IMPACT in temperature homeostasis.

We have previously provided several lines of evidence that IMPACT is an inhibitor of GCN2 activity. The data shown here add further evidence for this role. On the other hand, the observations reported here in animals lacking both IMPACT and GCN2, together with previous results from this group, strongly suggest the existence of GCN2-independent functions for IMPACT. For example, IMPACT promotes neurite outgrowth in a partially GCN2-independent manner [7]. In *C*. *elegans*, the lack of IMPACT results in a GCN2-independent larval arrest [23]. IMPACT and its yeast ortholog Yih1 interact with actin [2, 3, 6]. IMPACT/Yih1 interacts with CDK2/3 and the yeast counterpart Cdc28, and Yih1 was shown to regulate the progression of the yeast cell cycle in a GCN2- and GCN1-independent manner [27]. This effect depends on residues located in the RWD domain, involving the same residues that were found to be important for the binding to GCN1 and to actin [2, 27]. It is likely then that the RWD region of the IMPACT/Yih1 protein may have additional functions besides GCN1-binding. The role of the "ancient domain" of IMPACT/Yih1 is not clearly determined, but it is required, although not sufficient, for actin-binding [2].

The data suggesting that IMPACT/Yih1 functions in cell cycle regulation and that it binds actin may bear relevance to its increased abundance during neuronal cell differentiation in cultures as well as during the development of the mouse brain [7]. The establishment of neuronal connections may be affected in mice lacking IMPACT, resulting in the phenotypes described here that are independent of GCN2. The study of animals lacking IMPACT opens new venues for the study of this highly conserved and developmentally regulated protein and for further understanding the role of eIF2 phosphorylation in mammalian physiology.

## Supporting information

S1 Text (DOCX)

S1 Fig Sleep architecture. **(A)** Daily distribution of sleep states in wild-type and *Imp*-KO mice. Values are in mean ± SEM in percentage. **(B)** Number of epochs for each sleep state in wild-type (n = 9) and *Imp*-KO mice (n = 4). Values are in mean ± SEM in the number of occurrences. **(C)** Mean duration of epochs for each sleep state in wild-type and *Imp*-KO mice. Values are means ± SEM in seconds. P>0.05 between groups in the three analyses (Student’s t-test).
(EPS)

S2 Fig Home cage activity. Values are means ± SEM of total distance measured for each group (n = 6 of each genotype) in four repeats of 12-hour each (9:00 a.m. to 9:00 p.m.). P>0.05 between groups (Kruskal-Wallis method).
(EPS)

S3 Fig Weight gain of mice lacking GCN2 maintained in a high-fat diet. Wild-type (WT) and *Gcn2*-KO animals (n = 10 for each genotype) were fed a high-fat diet (HFD) and weighted at the indicated weeks. Week zero indicates the weight of animals maintained in normal chow, before being transferred to the high-fat diet. P>0.05 between groups at all time points (Student’s t-test).
(EPS)
