# Supplementary material for: The GCN2 inhibitor IMPACT contributes to diet-induced obesity and body temperature control
Source: PLoS One. 2019 Jun 5;14(6):e0217287. doi: 10.1371/journal.pone.0217287 (PMC6550387; doi:10.1371/journal.pone.0217287)
Supplement: S1 Text — (DOCX) [file pone.0217287.s001.docx]

**Supporting Information**

**Material and Methods**

**Sleep studies**

4 male *Imp*-KO mice and 9 male wild-type littermates (3–4 month old) were surgically implanted with electrodes (nickel-chrome wire) for chronic recording of electrocorticographic (ECoG) and electromyographic (EMG) activities. Briefly, after mice were anesthesized with ketamine (70-80 mg/kg) and xilazine (9 mg/kg), two pairs of bipolar electrodes were implanted ipsilaterally (ML = 2,0 mm and AP= -2,0 mm; ML = 2,0 mm and AP = +3,0 mm relative to Bregma) for ECoG, and one pair of bipolar electrodes was implanted into the cervical muscles for EMG. The electrodes were fixed to the cranium with acrilic cement. After surgery, animals were placed in individual cages for a 5-day recovery. After 2 days of habituation to the recording cables, ECoG and EMG recordings were initiated and proceeded for four days, with the aid of the Somnologica software (EMBLA Medical Digital Polygraph). Food and water were available *ad libitum*.

Sleep recordings were divided into 10-second epochs and scored blindly as Wakefulness (WAK), Slow Wave Sleep (SWS) or Rapid Eye Movement Sleep (REM), through visual and digital analysis performed with MATLAB (Mathworks). Wakefulness was characterized as a low-voltage and high-frequency ECoG signal with increased and variable EMG. Slow Wave Sleep was defined by high-voltage and low-frequency ECoG signal and low regular muscular tone in the EMG. Rapid Eye Movement was identified by regular theta waves (5–7 Hz) in the ECoG with absence of muscle tone in the EMG.

Sleep parameters included the following: Percentage distribution of sleep states, number of epochs for each sleep state, and mean epoch duration for each sleep state. A Student t-test was carried out for the analysis for each sleep parameter in order to determine differences between groups. A P value ≤ 0.05 was considered to be statistically significant.

No statistically significant differences were found between *Imp*-KO and wild type animals in any of the sleep parameters analyzed.

**Home cage activity measurements**

The subjects were brought to the behavior room 30 minutes before the test, for acclimatization. The animals were allocated in their original home cage, but the steel cover was substituted for a plexiglass cover for optimal image capture. During the test the animals had free access to chow but the water supply was removed for a better video performance. After the test, all subjects returned to the regular maintaining conditions. The test was conduct during 12 hours, starting at 9 a.m. and finishing at 9 pm. The images were tracked in EthoVision® software, and ambulation was registered in centimeters. The activity was registered in four consecutive days. For analysis, the period was divided into three set points, at four-hour intervals (9am-1pm; 1-5pm; 5-9pm).
